# Sex‐Specific Ultraviolet Radiation Tolerance Across *Drosophila*

**DOI:** 10.1002/ece3.70985

**Published:** 2025-02-25

**Authors:** James E. Titus‐McQuillan, Brandon A. Turner, Rebekah L. Rogers

**Affiliations:** ^1^ Department of Bioinformatics and Genomics University of North Carolina Charlotte North Carolina USA

**Keywords:** adaptation, *Drosophila yakuba*, natural selection, São Tomé and Príncipe, speciation

## Abstract

The genetic basis of phenotypic differences between species is a longstanding question in evolutionary biology. How new genes form and selection acts to produce differences across species are fundamental to understanding how species evolve. Adaptation and genetic innovation arise in the genome from a variety of sources. Functional genomics requires both genetic discoveries and empirical testing to observe adaptation between lineages. We explore two species of *Drosophila* from the island of São Tomé and mainland Africa, *D. santomea* and *D. yakuba*. These two species have varying distributions based on elevation on São Tomé, with populations of *D. yakuba* also inhabiting mainland Africa. Genomic/genetic evidence shows genes between species may have a role in adaptation to higher UV tolerance. We conducted empirical UV assays between *D. santomea* and both *D. yakuba* populations. Flies were shocked by UVB radiation for 30 min on a transilluminator apparatus. Custom 5‐wall acrylic enclosures were constructed for viewing and containment of flies. Island groups show significant differences between fall‐time under UV stress and recovery time post‐UV stress test between populations and by sex. This study shows evidence that mainland flies are less resistant to UV radiation than their island counterparts. Differential expression analysis also shows potential for new mutations and local adaptation for DNA repair of *D. santomea*. Understanding the mechanisms and processes that promote adaptation and testing traits within the context of the genome is crucially important to understand evolutionary machinery.

## Graphical abstract

This study investigates the genetic basis of phenotypic differences between two species of *Drosophila*, *D. santomea* and *D. yakuba*, focusing on their adaptation to UV tolerance and stress. Genomic and empirical evidence shows that island populations of *D. santomea* have developed greater resistance to UV radiation compared to their mainland counterparts, with significant differences observed in UV stress recovery times. These findings highlight the importance of local adaptation and genetic innovation in evolutionary biology in island systems.

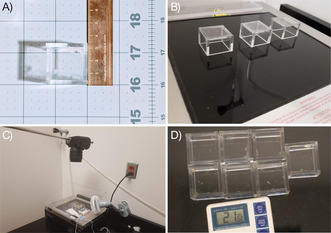

## 1 Introduction

The evolutionary response to shifting selective pressures is among the most profound open questions in evolutionary theory (Darwin 1859; Dobzhansky 1937). How organisms develop novel phenotypes allowing them to invade new habitats or survive under environmental shifts is essential to understand the outcomes and trajectory as organisms respond to new selective regimes. If adaptation can follow only a few paths to survival, then we expect species to show high rates of convergent evolution (Stern 2013). However, if multiple different modes of phenotypic change allow survival in the face of environmental shifts, then rates of convergence may be lower (Emery and Clayton 2004; Whittall et al. 2006; Zhou et al. 2008; Reviewed in Rosenblum et al. 2014). In such a case, the existence of multiple paths to success may allow more species to invade open niches more readily than if all must follow a single phenotypic or genetic solution (Kauffman and Levin 1987; Schoville et al. 2012). Species that harbor greater amounts of standing genetic and phenotypic variation may be more adept at invading open niches than species with limited variation (Hermisson and Pennings 2005). In theory, readily available standing variation can serve as an instant reservoir of phenotypic and genetic diversity, accessible to species undergoing selective shifts.

**FIGURE 1:**
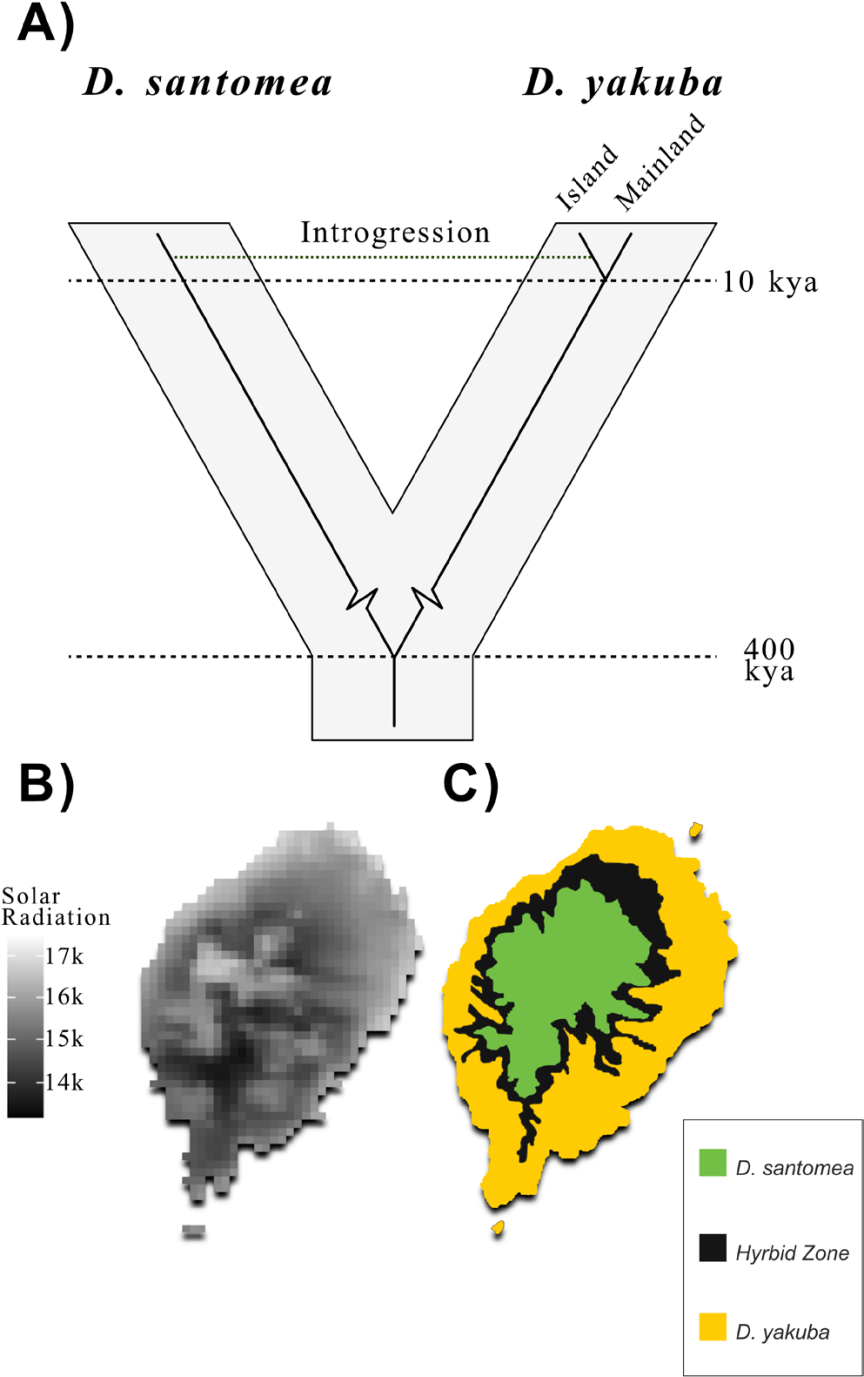
Phylogeny and regional maps broadly illustrating the relationships between populations in the *D. santomea*/*D. yakuba* across the island of São Tomé. (A) A toy rendition of the phylogentic relationships between *D. santomea* and *D. yakuba* through time. (B) Solar radiation data map constructed from WorldClim 2.1 historical climate data of the island of São Tomé. It is an overlay among all months for a holistic annual solar radiation São Tomé is exposed to, with lighter colors signifying higher solar radiation (kJ m^−2^ day^−1^) and darker colors delineating less solar radiation. (C) Estimated ranges between *D. santomea* and *D. yakuba* (adapted from Lachaise et al. 2000).

By using island systems to study the evolutionary response to discrete shifts in habitat, we can observe the phenotypic and genotypic response to environmental changes (Reviewed in Brown et al. 2013). Of particular interest is the island of São Tomé, where two species of *Drosophila* derived from the same progenitor population on the mainland have invaded new island habitats (Figure 1) (Lachaise et al. 2000). This species complex forms a natural experiment in island invasion, where we can use populations of *D. yakuba* residing throughout mainland Africa as a ‘control group’ to understand how phenotypes have changed on the island. *Drosophila santomea* invaded approximately 400,000 years ago (Figure 1A) (Llopart et al. 2002) and now is considered an island endemic not found below 1150 m on the volcano Pico de São Tomé [2024 m] (Lachaise et al. 2000). *D. santomea* is found in mist forests with lower temperatures (Llopart et al. 2002; Matute et al. 2009). *D. yakuba* invaded the island again in a secondary colonization event, around 10,000 years ago offering a second case of habitat invasion from the same mainland ancestral population (Figure 1A) (Cariou et al. 2001; Coyne et al. 2002; Obbard et al. 2012). *D. yakuba* is found in disturbed, mesic, or secondary forest habitat at lower altitudes, at a partially overlapping range with *D. santomea* (Figure 1) (Lachaise et al. 2000; Cariou et al. 2001). *D. yakuba* and *D. santomea* species show partial, but not complete, reproductive incompatibilities where introgression is common in the overlapping portion of their range (Figure 1) (Lachaise et al. 2000; Cariou et al. 2001; Coyne et al. 2002; Llopart et al. 2002; Obbard et al. 2012; Matute et al. 2009; Matute and Harris 2013).

Modern *D. santomea* have reduced abdominal pigmentation compared to ancestral *D. yakuba* populations, even along elevational gradients (Lachaise et al. 2000; Llopart et al. 2002; Matute and Harris 2013). While *D. yakuba* inhabits São Tomé's lower elevations; the species is not found above 1400 m, and possesses heavily pigmented abdominal segments among both sexes, with darker phenotypes increasing in frequency as elevation gradients increase (Matute and Harris 2013).

### 1.1 Local Adaptation to UV Stress

*D. santomea*, possessing a paler phenotype despite its higher altitudinal range, is curious, as it contradicts expectations for high‐altitude adaptation and other known solutions for survivorship under increased UV exposure, such as becoming melanistic. Melanism is a well‐studied mechanism, typically helping organisms combat harmful ultraviolet radiation and thermoregulate. It is commonly observed in many metazoan groups—pocket gophers (Goldman 1947), lizards (Reguera et al. 2014), grasshoppers (Harris et al. 2013), wasps (de Souza et al. 2020), and even zooplankton (Ulbing et al. 2019). Even in other species of *Drosophila*, high levels of melanin may be a trait that confers ultraviolet resistance at higher elevations (Pool and Aquadro 2007; Zhao et al. 2015). Matute and Harris (2013), too found that *D. yakuba* increases pigmentation at high elevation, but *D. santomea* does not. *Drosophila* with melanistic phenotypes increasing in frequency, due to increased ultraviolet exposure, is observed in mainland Africa along latitudinal gradients (Bastide et al. 2014). Though Rajpurohit and Schmidt (2019) found that pigmentation contradicts latitudinal ultraviolet radiation exposure. Davis and Moyle (2019) also did not find that darker pigmentation was a trait that correlated to more exposure to ultraviolet radiation in the *Drosophila americana* complex. Previous work empirically testing ultraviolet tolerance on latitudinal clines has shown that ultraviolet radiation has a significant effect on survivorship in *D. melanogaster* embryos (Svetec et al. 2016). Increasing melanin production confers protection from ultraviolet radiation by acting as an absorbent filter preventing penetration and subsequent DNA damage (Brenner and Hearing 2008). This “altitude‐induced melanism” is observed commonly across diverse taxa, yet not in *D. santomea*.

Prior experiments have shown that short‐term ultraviolet exposure reduces longevity in the *D. yakuba—D. santomea* island complex, but with lesser effects in the altitude adapted *D. santomea* (Matute and Harris 2013). These experiments proposed a model that stress responses in high altitude adapted flies confer beneficial effects in survivorship after ultraviolet exposure (Matute and Harris 2013). There was a noted negative correlation between pigmentation and ultraviolet stress contrary to previous expectations (Matute and Harris 2013). New work using population genomic comparisons between mainland and island populations has revealed signatures of local adaptation in *D. santomea* (Turner et al. 2021). These whole genome scans of selection have adaptive rearrangements that induce gene expression changes at three different loci in the ultraviolet repair machinery (Turner et al. 2021). These new genes‐up approaches with direct comparison to mainland ancestral populations suggest an intriguing possibility of ultraviolet resistance through a distinct mechanism from the previously proposed stress response or lack of pigmentation. To better understand how *Drosophila* may have adapted in these island environments, controlled, lab‐based phenotyping screens can help connect new information on the evolution of genotypes to modes of evolution for phenotypes in *D. santomea* and *D. yakuba*.

### 1.2 Experimental Design

Comparing the original progenitor population on the mainland and these two separate island populations (Figure 1A), we can establish the instance and magnitude of phenotypic changes in addition to the current phenotypic state on the island. These surveys, along with comparisons to the island's original populations, can help us determine whether low‐frequency standing variation for UV resistance in mainland populations facilitates immediate adaptation to new niches or if new phenotypes develop independently in island environments. With two independent waves of island invasion, we can assess whether *D. santomea* and island *D. yakuba* exhibit similar phenotypic changes compared to their mainland counterparts. Hence, these experiments can determine whether these two independent island adapted populations might have similar adaptive trajectories and fully convergent evolutionary outcomes. Alternatively, we can explore whether these two locally adapted populations show key differences in their phenotypes that suggest parallel but independent modes of adaptation. Our experimental precision and expanded comparisons with mainland populations can help to relate phenotypic change to fundamental models of evolution and the role that reservoirs of standing variation may play in adaptive change.

This study aims to determine how phenotypes have shifted among locally adapted island populations on São Tomé, particularly through comparisons with ancestral mainland populations. Previous experiments focused on short‐term ultraviolet exposure, partly because extended UV exposure raises temperatures, introducing the risk of confounding variables like thermal shock, which sometimes necessitates additional experiments to account for these effects (Matute and Harris 2013; Svetec et al. 2016). New experimental designs are necessary to gain a clearer view of morbidity as well as mortality during longer term ultraviolet exposure. To facilitate experiments in ultraviolet tolerance for *Drosophila*, we developed a new apparatus that enables high‐throughput surveys in batches of flies. It allows for implementation of video recordings, improving reproducibility and rigor, while controlling confounding variables and minimizing heat shock. This new apparatus enables surveys of acute ultraviolet exposure with greater precision to understand the direct and immediate effects of ultraviolet strain. Our ultraviolet tolerance assays complement previous findings, revealing a new axis of phenotypic change in response to shifting environmental pressures from ultraviolet exposure. These assays allow us to observe the direct effects of ultraviolet exposure without the risk of confounding or interacting variables that may appear over the longer lifespan of the fly.

### 1.3 Assays to Reveal Sex‐Specific Traits

The *D. yakuba* – *D. santomea* species complex is known to house sex‐specific divergence from the ancestral mainland populations for several traits. Males of *D. santomea* are paler than *D. yakuba*, with little to no pigmentation (Llopart et al. 2002), a key trait used to distinguish species from one another. In contrast, females show more modest differences with reduced pigmentation at stripes along abdominal segments and the pigmentation spot on the lower abdominal bands (Llopart et al. 2002). Additionally, there is a known size asymmetry for males and females in *D. yakuba* and *D. santomea* (Llopart et al. 2002). Temperature is also sex‐specific (Llopart et al. 2005a, 2005b), as *D. santomea* female fertility is markedly lower than males at higher temperatures (Matute et al. 2009). Moreover, emerging genetic studies also observe signals of the large‐X effect within the *D. yakuba*—*D. santomea* complex resulting in speciation (Llopart 2012). This complex confers Haldane's Rule of sterility (Haldane 1922; Coyne 1985) through prior studies showing the X chromosome has a disproportionately large effect on hybrid male sterility (Coyne and Orr 2004; Moehring et al. 2006a, 2006b), which may be correlated with the rapid divergence of sex‐specific traits.

In this study, our assays include tests of morbidity and mortality for both males and females, allowing us to determine whether phenotypes evolve in parallel for both sexes. Comprehensive assessment of phenotypic changes by sex is all the more imperative given the sex‐specific divergence and large‐X effects for other traits in *D. yakuba* and *D. santomea* (Llopart 2012). Because evolutionary modes, reproductive strategies, and molecular or biological backgrounds differ in the sexes, it is imperative to include sex as a biological variable in phenotypic analyses (Lee 2018). As we report, this use of sex as a biological variable offers greater precision in our phenotypic assays and allows us to identify significant sex‐by‐species interactions that would be missed in single sex or mixed‐sex assays. Theory suggests that pleiotropic effects can result in antagonistic evolutionary pressures if traits have sex‐specific impacts on selection (Darwin 1871; Fisher 1931; Parker 1979; Lande 1980). Because different sexes employ alternative reproductive strategies, both risk tolerance and phenotypic outcomes may diverge between males and females (Trivers 1972; Lande 1980; Parker 1979; Bonduriansky and Chenoweth 2009). In these scenarios, a trait that may be beneficial in one sex could be detrimental in the other leading to antagonistic pleiotropy (Lande 1980; Rice 1987; Bonduriansky and Chenoweth 2009; Rowe et al. 2018). Sexually dimorphic traits, however, can remove or reduce such constraints as females and males may adapt to their behavioral and reproductive environments independently (Trivers 1972; Lande 1980; Albert and Otto 2005). This freedom from antagonistic sex‐biased constraints may be even more important if evolutionary risks experience different payouts in males and females (Trivers 1972). Without the inclusion of sex as a biological variable, phenotypic differences might be obscured, leading to false impressions of similar evolutionary parallelism across species. With sex‐specific assays, we can better capture the full spectrum of phenotypic divergence during adaptation under environmental shifts. Hence, these types of assays that include sex as a biological variable are essential to fully understand both the modes and outcomes of evolutionary processes.

## 2 Methods

### 2.1 *Drosophila* Lines

For this study, we used a total of 24 inbred isofemale lines consisting of two species spanning the island São Tomé and mainland Africa. The experiment consisted of 9 *Drosophila santomea* lines and 15 *Drosophila yakuba* lines between São Tomé (five lines) and mainland *D. yakuba* (10 control lines). Fly lines from the island of São Tomé were provided by the Matute lab from his previous field collections (Comeault et al. 2016; Turissini and Matute 2017; Cooper et al. 2017) and reported in Rogers et al. (2014). Our mainland *D. yakuba* control wild‐type lines were provided by the National *Drosophila* Species Stock Center, originally deposited as stocks 14,021–0261:38–14,021‐0261:51. The control stocks used in this study are as follows, with old stock names being used and center stock names in brackets: CY21B3 [Nguti, Cameroon 2], CY28A [Nguti, Cameroon 4], NY62 [Nairobi, Kenya 2], NY65 [Nairobi, Kenya 3], NY73 [Nairobi, Kenya 4], NY81 [Nairobi, Kenya 5].

### 2.2 Sample Collection

For each isofemale line, we generated experimental animals by allowing groups of parentals (around 10–20) to mate and lay eggs in fresh vials, containing 4 mL of standard food, placed into an incubator at 22°C and 24°C for *D. santomea* and *D. yakuba*, respectively. The incubator was programed with a 12:12 light: dark cycle and 50% humidity. Emerging virgin offspring were anesthetized with CO_2_ in the morning and placed in new vials with standard media, filtered by sex for each line. They were then allowed to mature for 3 days prior to experiment without dry baker's yeast, to prevent flies potentially becoming stuck, under the same incubator conditions above.

### 2.3 Enclosure Design and Construction

To view the ultraviolet tolerance of flies for UV trials, we constructed custom enclosures made of clear acrylic. Acrylic, as a material, is well suited for these experiments as it has superlative clarity and is easily manipulated, unlike other materials such as glass. We cut four identical side panels and one top panel with an open bottom out of a 3 mm thick sheet on clear acrylic using a GlowForge© laser cutter. Panels are fused to form the enclosures using Weld‐On 3 solvent cement, a clear acrylic adhesive that retains full visibility during UV assays. The interior volume of the enclosures is 26 mm wide by 26 mm long by 19 mm high. For full schematics see Supporting Information (enclosure_schematic.pdf [for laser cutting importation] and enclosure dimensions).

### 2.4 Experimental Design

UV exposure was generated on an Analytik‐Jena 8‐watt UVP 3UV Transilluminator LMS‐20 [95‐0417‐01 (US)]. We set the light spectrum to 302 nm (UVB). The transilluminator was given time to warm up before conducting the experiment for around 15–25 min as flies were gathered. Our transilluminator has internal cooling, though the platform can still heat up. To mitigate any form of heat shock, we monitored the platform with a temperature probe and cooled the platform with a fan. Extra care was taken with setting up the cooling so as not to have flies influenced by the air circulation or blown off the UV platform. One key factor to eliminating air flow as a variable was to design and fabricate enclosures [see above, *Enclosure Design and Construction*]. Considering that *D. santomea* inhabits relatively cooler temperatures (Lachaise et al. 2000), previous studies on UV tolerance have encountered limitations due to the necessity of separately assessing additional variables such as heat and desiccation alongside the primary experiment (Matute and Harris 2013; Davis and Moyle 2019; Svetec et al. 2016). In our own assays, without cooling, UV exposure resulted in thermal shock from 25.1°C to 34.3°C in 30 min, near sterilization risk threshold or even lethality for *Drosophila* (David et al. 2005). This thermal stress is therefore a serious limitation that in the past has prevented longer UV exposure and surveys of acute UV stress. To accomplish temperature regulation, we directed a fan on the glass transilluminator plate, providing directed airflow across the surface. Because glass is an insulator, heat does not easily penetrate, and providing ample airflow allows for heat to dissipate more rapidly from the apparatus. Temperature readings occurred in real‐time using a calibrated digital temperature probe. UVB incidence was measured 10 cm from the transilluminator platform after the warm‐up phase, per manufacturer's recommendation. Then taken at multiple areas with a Solarmeter Model 5.7 Sensitive UVA + B Meter. Flies were taken from the incubators and anesthetized. Groups of five flies, used as replicates, were placed in an open bottom enclosure made of clear acrylic. The open bottoms of the enclosures were essential to prevent inadvertently filtering UV light through the acrylic during exposures.

Video recordings were conducted on a Sony Alpha 6400–APS‐C Interchangeable Lens 24.2‐megapixel Camera. The camera was attached to a ball head mount on a C‐stand positioned approximately 2–3 ft above the transilluminator. We recorded at 1080p resolution in MP4 format using a Tamron 17‐70 mm f/2.8 Di III‐A VC RXD Lens for Sony E mount. The field of view (FOV) was set so that all enclosures were in view and *Drosophila* specimens were in focus. To mitigate shaking and preserve image quality, without touching the camera while recording, we used the Sony Imaging Edge Mobile application. This allowed for remote viewing on a mobile device and remote operation of camera functions without the need to touch or disrupt the recordings. This function reduced the need for active hands‐on personnel. The result was an assay that decreased the personnel hours required for experiments, enhanced safety by preventing UV exposure to the researcher, and minimized unexpected incidents that could disrupt the recording of assay screens.

Our goal was to examine morbidity and mortality immediately resulting from UV stress, without the risk of interactions with other confounding variables (e.g., pathogen risk, reproductive effects) over the longer lifespan of the flies. We performed 24 trials with 5 replicates of 5 flies for females, and 24 trials with 5 replicates of 5 flies for males. The maximum number of replicates per trial was 6 for strains NY73, Cascade 19.16 females, and Thera2005 males. Flies were exposed to an average UV incidence of 1849 μW/cm^2^ for 30 min, recorded 10 cm away from the UV platform. The trial recordings lasted for approximately 30 min of video with a total file size of ~3.0 GB of mp4 files, which was later reduced to approximately 200–300 MB after processing in Adobe Premiere Pro CC. Video logs were then observed to determine when a fly fell due to UV exposure. The time when a fly fell was recorded to the second. A fly that fell to UV exposure was defined as a fly that had fallen off its feet and remained motionless for at least 5 s. Sometimes flies may twitch or buzz. They may also get up and walk for a time before falling over again. The first consistent fall without recovery was tracked for each individual fly and recorded. Out of all trials, 121 replicates for males and 122 replicates for females were scorable from video footage.

Flies were collected and monitored post‐UV activity and categorized by recovery status. Status is categorized by active, barely active, inactive, and dead. Active is defined by a fly specimen that is actively moving around and could be flying. This category of fly is not ostensibly discernable from a fly not under any stress. A barely active fly is moving but highly lethargic compared to a fly under normal conditions. A fly will be slow, may fall while mobile, and, while active, is not fully mobile. Inactive flies are not mobile; however, their bodies will have some source of life such as twitching appendages. Usually, an inactive fly will show signs of activity given external stimuli, for example, tapping the vial will yield slight twitches and movement. These flies are not motile. Finally, the “dead” category is defined as a fly that is no longer showing any discernable activity, without movement even when given an external stimulus. Post‐UV activity was recorded using a dissecting scope at 30 min, 1‐h, 2‐h, 4‐h, and 24‐h time intervals after filming.

### 2.5 Geographic Reconstruction of Solar Stress

Solar radiation map of the island of São Tomé was constructed using WorldClim 2.1 historical climate data (Fick and Hijmans 2017), taking solar radiation raster layers at 30 s resolution. All raster layers were clipped by a mask, a shape file of the administration zone of São Tomé and Príncipe. In total, 12 layers (corresponding to each month) were clipped by the shape file. Geo TIFF files were then converted to ascii files and overlaid to display a holistic annual solar radiation (kJ m^−2^ day^−1^) of São Tomé displayed in Figure 1, where lighter color signifies higher solar radiation values. All clipping functions and raster translations were conducted in QGIS π (v. 3.14.1‐Pi). The range map of *D. santomea* and *D. yakuba* was adapted from Lachaise et al. (2000) using vector graphics in Adobe Photoshop CC and Illustrator CC.

A maximum entropy analysis [Maxent] (Phillips et al. 2017) for a species distribution model was also conducted, using eight *D. santomea* and nine *D. yakuba* localities from Comeault and Matute (2021). The sampling size was too small to make meaningful niche space predictions, but it can be found on our Zenodo (DOI: 10.5281/zenodo.13894098).

### 2.6 Statistical Analysis

All statistical analyses were conducted in R v4.3.1 (R Core Team 2023) using RStudio 2023.09.0 Build 463 (RStudio Team 2023). For our real‐time fly activity under UV stress, statistical tests were conducted among and between factors sex and population ancestry and their interaction. We used a Wilcoxon test (Bauer 1972; Hollander and Wolfe 1973) independently on male and female flies to compare whether groups of flies originating from different geographic regions are significantly different. We used the pairwise_test() function from the rstatix package (Kassambara 2023). An ANOVA was conducted on three factors—sex, population, and the interaction between sex and population. After variance analyses, we conducted Tukey's Honestly Significant Difference (HSD), with the TukeyHSD function (Miller 1981; Yandell 1997), post hoc tests on the paired means from our ANVOA results for the three factors above. We tested samples for unequal variance using an *F*‐Test. To understand if there are random effects that different fly lines may have on the model, we used a Linear mixed model fit using Satterthwaite's method from the package lmerTest function lmer4() (Kuznetsova et al. 2017) using fly lines as the random effect.

This study also recorded post‐UV exposure activity. We conducted a MANOVA (Hand and Taylor 1987; Krzanowski 1988) to test differences between categories of fell status (active, barely active, inactive, dead) across activity time, fly population, and sex using the manova() function in R. To test the interaction of activity fell time (real‐time UV exposure tolerance) to recovery (post‐UV activity) we used a general linear model using Satterthwaite's method for male and female flies, independently, to test the interaction of UV tolerance and recovery across populations.

### 2.7 Differential Expression Analysis

To determine whether there might be genetic changes underpinning the empirical UV exposure results, we used previously generated RNA sequence data for 5 lines of *Drosophila* overlapping this study (full methods available in Turner et al. 2021). Flies were reared in a temperature‐controlled incubator, and virgin adults were aged 5–7 days post eclosion. For each line, we extracted the gonads and flash froze tissues in liquid nitrogen for both the gonads and soma (carcass without gonads) using 5 replicates for each sex and tissue. In brief, RNA extraction was conducted using Zymo DirectZol RNA Microprep (Zymo Research). Libraries were prepared manually following the manufacturer's protocol (TruSeq Stranded mRNA LS, RevD; Illumina). Sequencing was conducted on an Illumina HiSeq 4000 platform using the 150 bp paired end (PE) Cluster Kit. For a more complete description, refer to Turner et al. (2021).

To evaluate differential expression in the samples, we used the program CuffDiff v2.2.2 (Trapnell et al. 2013) to establish *p*‐values correcting for multiple testing and FPKM for each tissue. For any gene that showed significant differential expression in a sample, we conducted functional gene annotation analysis with DAVID v2023q1 (Huang et al. 2009). *D. yakuba* genes were matched to their orthologs in *D. melanogaster* using Flybase (Drysdale and the FlyBase Consortium 2008). Genes that had no orthologs in *D. melanogaster* were excluded from gene ontology analysis. Using these orthologs, we tested for overrepresented gene functions and generated high‐throughput functional annotations with the low‐stringency option in DAVID. In addition, to ensure complete representation of genes relevant to UV tolerance, we obtained a list of known UV tolerance candidate genes from Svetec et al. (2016). From the combined list, we identified genes from our genome‐wide screen for expression changes that were also associated with functional categories such as DNA repair, stress tolerance, and UV tolerance. From this pool of candidate genes, we then focused on those with functions most likely to influence the phenotypic assays described above.

## 3 Results

### 3.1 Solar Radiation and Distribution Map

Estimates of higher solar radiation (kJ m^−2^ day^−1^) concurred with the distribution of *D. santomea* (Figure 1) at higher elevations and higher solar radiation. *D. santomea* also, potentially, inhabits certain areas along hillsides with low solar radiation (see cursory Maxent analysis [Zenodo]). Though it should be noted that solar radiation and UV radiation do not have a 1:1 correlation (International Agency for Research on Cancer 2012). A lack of solar radiation does not necessarily equate to lower ultraviolet exposure; in fact, UV levels are expected to be greater at higher altitudes. Lachaise et al. (2000) described the habitat of *D. santomea* as a species that is found in mist forests, while *D. yakuba* is found in more disturbed areas (Llopart et al. 2005a). Current evidence suggests that *D. santomea* experiences higher UV exposure across its range. Unless currently unidentified physical solutions exist to shelter flies from UV, we expect radiation stress to be greater for this higher altitude species.

### 3.2 Apparatus Function

The main purpose of our study was to establish whether there were differences in ultraviolet resistant phenotypes across *Drosophila* species on São Tomé compared to mainland Africa. In pursuit of this goal, we have developed new methods and a new apparatus to assay UV tolerance. The design and function of our new phenotyping setup enhance reproducibility and rigor through recorded video, improve control over confounding variables like temperature, reduced the need for hands‐on time from lab personnel, and increase safety by minimizing the risk of UV exposure. We recommend the use of similar designs for future studies on ultraviolet experiments. In addition to standard method descriptions, schematics for construction are included here for the benefit of the greater scientific community as well as for reproducibility (see Supporting Information). We hope this method's release allows for community driven innovation to modify and improve on the features presented.

Here we generated 1080p MP4 codec video files to observe the UV tolerance of flies from the island of São Tomé between island *D. yakuba* and *D. santomea*, with mainland *D. yakuba* fly lines used as controls. Average temperature during trials ranged from 21.9°C to 23.4°C (minimum temperature 19.7°C and a maximum temperature 29.7°C, depending on ambient room temperature), reducing changes of heat shock during UV stress—a key feature built into the apparatus. Without mechanisms for airflow, temperature could fluctuate 9.2°C or more in 30 min of UV exposure, risking confounding effects of heat shock that can occur rapidly in *Drosophila* (O'Brien and Lis 1993). Hence, our experimental setup enables longer UV exposure than past experiments and finer control of entangled variables.

**FIGURE 2:**
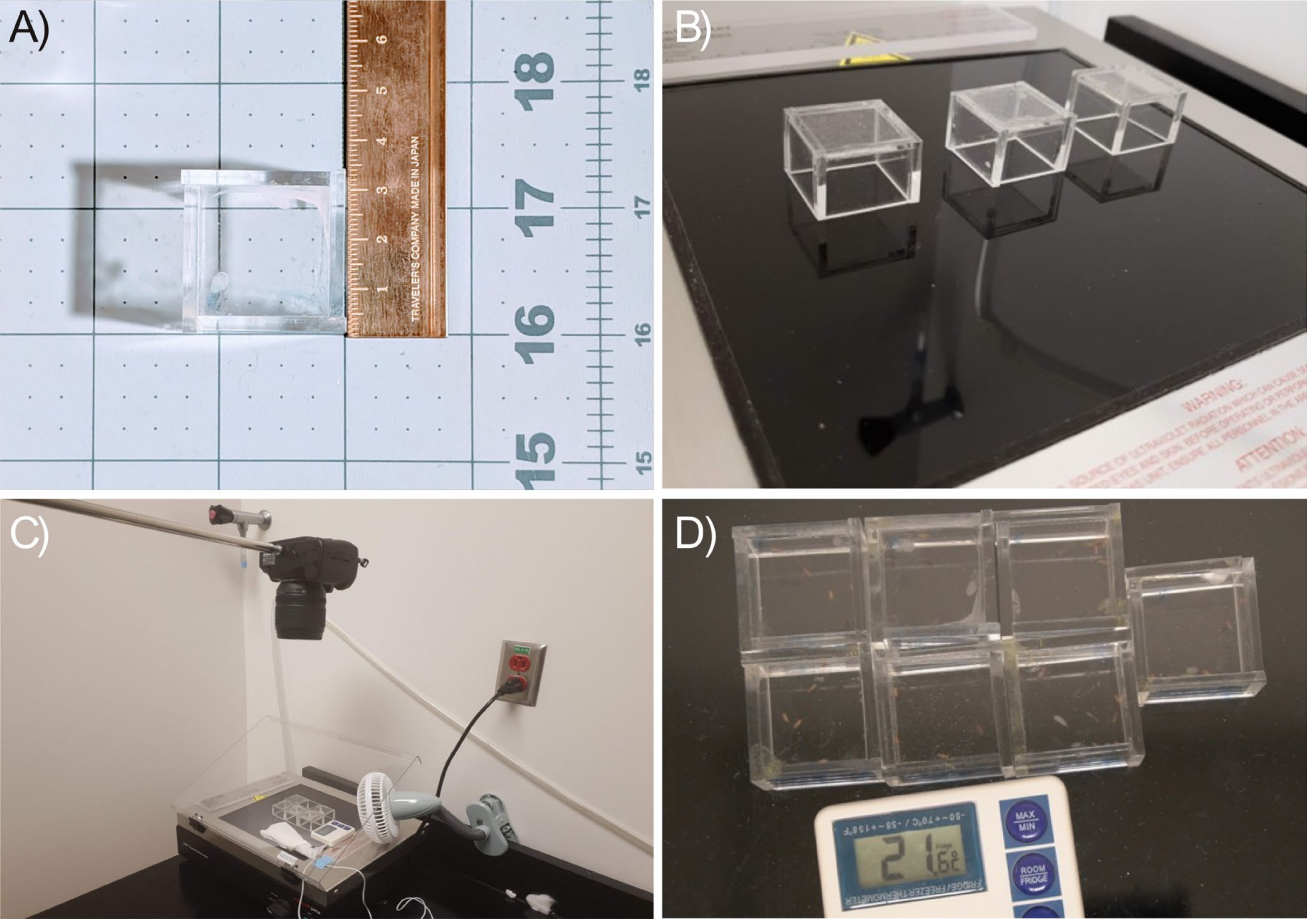
Recording apparatus set‐up. We have developed a new apparatus that allows for surveys of ultraviolet tolerance safely, efficiently, and with improved control of confounding variables. (A) Fully assembled enclosures to contain fly strain replicates. (B) Enclosure placement on the UV platform. Multiple enclosures allow for fly strains to be surveyed in batches within the camera field of view. (C) Full set‐up of apparatus with cooling, temperature probe, camera, and enclosure placement. Video recordings reduce hands‐on researcher time and improve reproducibility and rigor for phenotyping assays. (D) Field of view (FOV) with flies contained on UV transilluminator platform with temperature probe read out visible in an active experiment. Temperature has been a confounding variable and concern in past UV tolerance assays. The ability to control heat in our apparatus improves accuracy. It further simplifies experimental design as it does not require secondary assays of temperature without UV exposure.

The full set‐up of the apparatus uses commonly found equipment in a biological laboratory. The apparatus set‐up and enclosure construction can be found in our experiment in the methods. Our methods, assemblages, and construction can be modified to suit any lab space as needed. Given the enclosures are made from easily manipulatable acrylic, air‐ports can be used to inject CO_2_ directly into an enclosure if an experiment requires anesthetization before enclosures are pulled. Here the function of our innovative apparatus serves four purposes: containment, specimen recovery, digital recording, and a platform for interaction of an external force to test a phenotypic trait. We consider this apparatus to be novel in both its description and its use. The platform may be modified to a particular external phenomenon being addressed. This setup improves reproducibility and rigor in UV studies as videos are available for review, and UV exposure and temperature are finely controlled during exposure. Furthermore, remote filming allows for a much safer work environment, where the operator does not need to be in front of the apparatus potentially exposing themselves unnecessarily to ultraviolet radiation. To our knowledge, we have not seen an apparatus like the one used in this study (Figure 2). Schematics are publicly available to facilitate similar studies in the field (see Supporting Information).

### 3.3 UV Tolerance Until Inactivation

**FIGURE 3:**
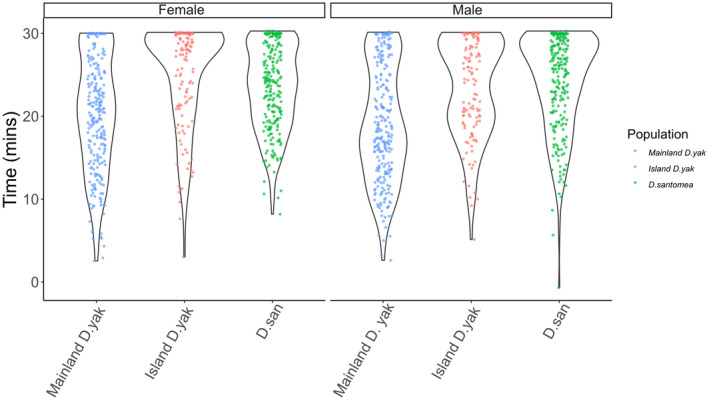
Violin plot of all flies during UV exposure separated by sex and population ancestry. Time in minutes of exposure when a fly fell during exposure. Greater UV tolerance is evident in longer times to fly inactivation. Females are the on the left with males on the right. Populations are separated by label on the x‐axis and by color with Mainland *D.yakuba*, Island *D. yakuba*, and *D. santomea* in light blue, orange‐red, and green, respectively. The time at which a fly falls is significantly different for the sex of the fly [*p* = 0.007] and its population [*p* = 0.000] (see Table 1). Populations on the island are, broadly, better adapted to ultraviolet exposure than the mainland population, with longer fall times having higher concentrations in the island populations, especially observed in *D. santomea*. The degree of variance for sex and population both are significantly different [Sex:Population ‐ *P* = 0.000].

Across all flies, we observed variation in UV tolerance depending on the population ancestry of flies as well as sex‐specific variation. There is a significant difference between sexes for UV tolerance, the time a fly fell due to UV exposure ([*p* = 0.007]; Figure 3; Table 1). Males had a broader distribution of tolerance, while females had a tighter distribution and are more UV tolerant (*F*‐test: [*F* = 0.90691, *p* = 0.007]).

**TABLE 1:** ANOVA of *Drosophila* sex and population with the Tukey honestly significant difference (HSD) test between sex‐population specific differences statistics on real‐time UV exposure.

	By sex‐population				
ANOVA	DF	Sum of squares	Mean sum of squares	*F*	*p*
Sex	1	126	126	7.123	0.00771
Population	2	4866	2433.1	137.579	***
Sex:Population	2	332	165.8	9.376	***
Residuals	1209	21,381	17.7		

	By sex‐population			
TukeyHSD	Difference	Lower	Upper	*p*‐adj
Male:Mainland Dyak‐Female:Mainland Dyak	−0.704866	−1.7127061	0.30297411	0.3449818
Female:Island Dyak‐Female:Mainland Dyak	4.1763135	2.9559909	5.39663605	***
Male:Island Dyak‐Female:Mainland Dyak	2.822634	1.5862518	4.0590162	***
Female:Dsan‐Female:Mainland Dyak	3.2173747	2.181647	4.25310177	***
Male:Dsan‐Female:Mainland Dyak	3.4907644	2.4610355	4.52049334	***
Female:Island Dyak‐Male:Mainland Dyak	4.8811795	3.6567433	6.1056157	***
Male:Island Dyak‐Male:Mainland Dyak	3.5275	2.2870574	4.76794259	***
Female:Dsan‐Male:Mainland Dyak	3.9222407	2.8816701	4.96281143	***
Male:Dsan‐Male:Mainland Dyak	4.1956304	3.1610298	5.23023108	***
Male:Island Dyak‐Female:Island Dyak	−1.3536795	−2.7721799	0.06482089	0.0713222
Female:Dsan‐Female:Island Dyak	−0.9589387	−2.2064294	0.28855188	0.2411661
Male:Dsan‐Female:Island Dyak	−0.6855491	−1.9280642	0.55696613	0.6153994
Female:Dsan‐Male:Island Dyak	0.3947407	−0.8684642	1.65794565	0.9484894
Male:Dsan‐Male:Island Dyak	0.6681304	−0.5901612	1.92642204	0.6542787
Male:Dsan‐Female:Dsan	0.2733897	−0.7883953	1.33517465	0.9775918

*Note:* Significant code: *p* = 0 delineated***.

There are significant differences in UV tolerance across these three populations of *D. yakuba* and *D. santomea* ([*p* < 0.001]; Figure 3; Table 1). At higher elevations, ultraviolet exposure is greater. A niche at higher elevation will have more UV stress than a niche at lower elevations (Figure 1). Our observations illustrated this between regions where UV tolerance is significantly different depending on population ancestry with *Drosophila* from island regions being more UV tolerant than the mainland, and populations at higher elevation also being more UV tolerant (Figure 3). The mainland control lines of *D. yakuba* were not as UV tolerant as island residents *D. yakuba* or *D. santomea*. Our Tukey HSD test showed that the mainland *D. yakuba* populations were significantly different from both island *D. yakuba* ([*p* < 0.001]; for all comparisons) and *D. santomea* ([*p* < 0.001]; for all comparisons) but were not significantly different between the sexes from each other (See Table 1 for all P‐value comparisons). However, there are observable differences in both island *D. yakuba* and *D. santomea* in the distribution for when a fly became inactive through UV exposure given sex (Figure 3). We observed that populations are a significantly different [Wilcoxon Test: *p*‐adj < 0.001] when conditioned by sex, except for island *D. yakuba* and *D. santomea* males; [Wilcoxon Test: *p*‐adj = 0.434].

Among the interaction of sex and population ancestry there were significant differences between factors and their interactions (Figure 3; Table 1). Both sex and population were significantly different [*p* = 0.007 and *p* < 0.001], respectively, with the interaction between them being significantly different as well [*p* < 0.001]. HSD *post hoc* tests were used to determine which pair group means were significantly different from each other. We observed that there was no difference between sexes within the mainland *D. yakuba* [*p* = 0.345], *D. santomea* [*p* = 0.978], or island *D. yakuba* [*p* = 0.071]. Between mainland *D. yakuba* and island *Drosophila*, there is a statistical difference between all interactions (Table 1). We conducted a linear mixed model fit using Satterthwaite's method. There was no significant difference in post‐UV or the interaction of post‐UV time and population ancestry in female flies, but a significant difference in population (Table 2). While male flies were significantly different in all factors that involved *D. santomea* from the mainland *Drosophila* and their interactions (Table 2). Males did not have fixed effects among the fly lines that were different from the model [intercept, *p* = 0.094]. Females, conversely, had fixed effects given a fly line [intercept, *p* = 0.005] (Table 2).

**TABLE 2:** Type III ANOVA table conditioned by sex with random effects on fly lines with active flies post‐UV exposure.

Type III Analysis of Variance Table with Satterthwaite's method					
**Males**					
*Random effects*					
Groups	Name	Variance	Std. Dev.		
Fly lines	(Intercept)	0.6633	0.8144		
Residual		0.2956	0.5437		
Number of obs: 605, groups: experiment, 119					
*Fixed effects*					
	Estimate	Std. Error	df	*t*‐value	Pr(>|t|)
(Intercept)	0.206893	0.122626	126.335644	1.687	0.09403
Post‐UV Time	−0.006174	0.00385	483.531688	−1.603	0.10951
Island *D. yak*	0.27911	0.212395	126.335645	1.314	0.19119
*D. san*	1.299905	0.179064	125.852456	7.259	***
Post‐UV Time:Population—Island Dyak	0.005221	0.006669	483.531687	0.783	0.43411
Post‐UV Time:Population—Dsan	−0.015696	0.005562	483.531687	−2.822	**

Type III Analysis of Variance Table with Satterthwaite's method					
**Females**					
*Random effects*					
Groups	Name	Variance	Std. Dev.		
Fly Lines	(Intercept)	0.9401	0.9696		
Residual		0.3535	0.5946		
Number of obs: 610, groups: experiment, 121					
*Fixed effects*					
	Estimate	Std. Error	df	*t*‐value	Pr(>|t|)
(Intercept)	0.400589	0.143213	126.32144	2.797	0.005963
Post‐UV Time	0.003019	0.004169	485.929963	0.724	0.469347
Island *D. yak*	1.117103	0.246457	126.32144	4.533	***
*D. san*	0.782448	0.210347	126.110165	3.72	***
Post‐UV Time:Population—Island Dyak	−0.00949	0.007175	485.929963	−1.323	0.186541
Post‐UV Time:Population—Dsan	−0.010045	0.006089	485.929963	−1.65	0.099652

*Note:* Significant code: *p* = 0 delineated***, 0.001 delineated**.

### 3.4 Recovery Time After UV Stress

**FIGURE 4:**
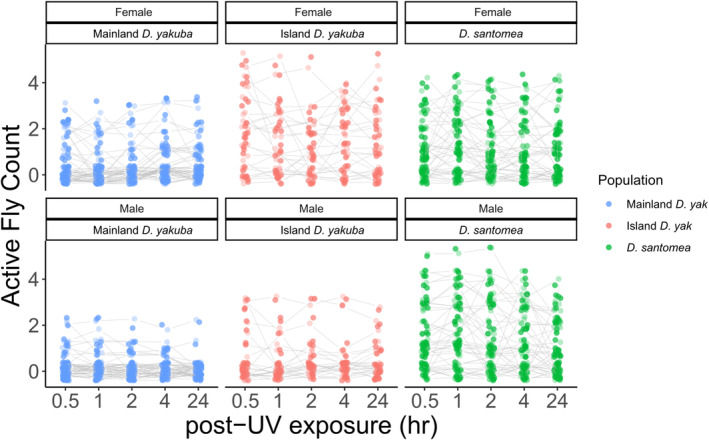
Split line plots of flies that are categorized as actively recovered partitioned by population ancestry and sex. Populations are separated by label on the x‐axis and by color with Mainland *D. yakuba*, Island *D. yakuba*, and *D. santomea* in light blue, orange‐red, and green, respectively. Each dot signifies a fly vial with the number of flies in the *Active* category on the y‐axis across each observed time point after UV exposure had ended. Females show better recovery than males post‐UV exposure. Island populations recover better post‐UV exposure compared to the mainland population.

**FIGURE 5:**
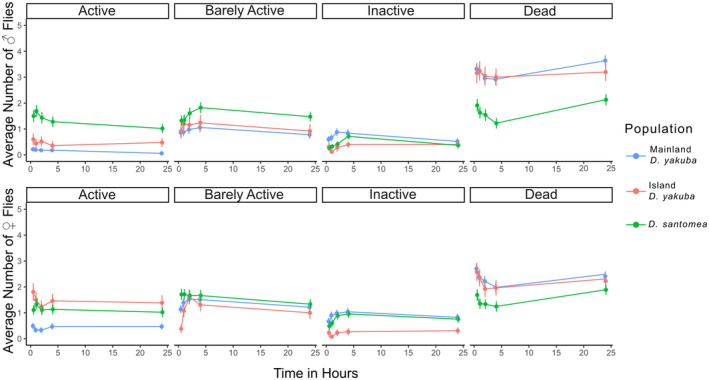
Line plots between males and females split by color based on population ancestry with Mainland *D. yakuba*, Island *D. yakuba*, and *D. santomea* in light blue, orange‐red, and green, respectively, with males on top and females on bottom. The average number of flies for each post‐UV exposure category with error bars (y‐axis) and time intervals post‐UV exposure (x‐axis). The x‐axis is time in hours from 30 min to 24 h post‐UV exposure. Across all categories island populations have better recovery to post‐UV exposure compared to the mainland. *D. santomea* males show the greatest recovery, while *D. yakuba* females have the most active flies post‐UV exposure.

In natural systems, direct mortality from UV exposure is expected to be costly, but morbidity may affect survivorship and reproduction as well. The duration of inactivation would increase chances of predation in nature and reduce efforts for foraging. We found that susceptibility to ultraviolet radiation during active UV exposure assays did not necessarily indicate lower recoverability, which was especially relevant given that *D. santomea* is not melanistic like other *D. melanogaster* conspecifics (Wittkopp et al. 2003a, 2003b; Matute and Harris 2013; Coolon et al. 2014; Svetec et al. 2016). UV recovery was observed across five‐time intervals after UV exposure assays concluded. There was a higher density of island constituent species having more active flies post‐UV exposure compared to that of the mainland *D. yakuba* lines (Figure 4). There were sex‐specific differences observed in female island *D*. *yakuba* ([*p* < 0.001]; Table 2) compared to the male island *D*. *yakuba* ([*p* = 0.191]; Table 2). Female island *D*. *yakuba* also had the highest survivorship among all three populations ([*p* < 0.001]; Figures 4 and 5; Table 2). *D. santomea* had high survivorship among these three populations between sexes ([*p* < 0.001]; Figures 4 and 5; Table 2).

We observed a higher propensity for female flies to recover more rapidly and efficiently than males across all populations. Among species, *D. santomea* has the highest recoverability. Across all groups by sex and population, female island *D. yakuba* have the highest recoverability ([*p* < 0.001]; Figure 5; Table 3).

**TABLE 3:** MANOVA results of recovery compared to sex, population, post‐UV time, and their interactions.

MANOVA						
	DF	Pillai	Approx. *F*	# DF	Density DF	*p*
Post‐UV time	1	0.007977	2.412	4	1200	0.04737
Population	2	0.227901	38.614	8	2402	***
Sex	1	0.04418	13.867	4	1200	***
Post‐UV Time:Population	2	0.004774	0.718	8	2402	0.67545
Post‐UV Time:Sex	1	0.002032	0.611	4	1200	0.65496
Population:Sex	2	0.055531	8.575	8	2402	***
Post‐UV Time:Population:Sex	2	0.002311	0.347	8	2402	0.94733
Residuals	1203					

*Note:* Significant code: *p* = 0 delineated***.

### 3.5 Interactions Between Mortality Versus Morbidity

To determine whether there is a difference between mortality vs. morbidity, we conducted a MANOVA comparing states of recovery by post‐UV time to sex and population. There was a significant difference between recovery state (response) and time of post‐UV exposure. Among our other treatments, there was a significant difference with sex and population and their interaction. There was an effect of when a fly faints from post‐UV exposure to the state of recovery post‐UV exposure [*p* = 0.047], though no significance with post‐UV interactions across populations, sex, and population*sex ([*p* = 0.675, *p* = 0.655, *p* = 0.947] respectively; Table 3). The *Pillai's* trace showed that population ancestry from which a fly was taken, as an independent variable, had the largest effect, followed by the interaction of population and sex, then sex.

### 3.6 DNA Repair Differential Expression

To gain a more comprehensive understanding of genetic changes in genes influenced by ultraviolet radiation, we conducted a genome‐wide survey of gene expression changes in each of the phenotyped isofemale lines. Prior genomic screens have revealed three structural variant mutations that induce gene expression changes in UV repair genes in *D. santomea* (Turner et al. 2021). To identify genetic changes that might potentially influence UV tolerance, we performed differential expression testing across populations for gonads and soma in adult flies. Gene expression analysis identifies several known UV tolerance genes or DNA repair genes with differential expression across populations. A list of significantly differentially expressed UV resistance genes are listed in Table 4.

**TABLE 4:** Table of candidate genes from our RNA‐seq genomic assays that have significant differential expression. This list of genes is from a DAVID functional clustering analysis focusing on DNA repair mechanisms.

GE number	Gene name	Abbreviation description	Line—Tissue	Log2 fold change
GE23311	*CycG*	Cyclin G (CycG)	Island Cascade‐1916—Testes	−0.483561
GE15960	*Pp2B‐14D*	Protein phosphatase 2B at 14D (Pp2B‐14D)	Thera2005—Ovaries	−0.296757
			Thera2005—Testes	1.30916
GE16425	*CG4078*	Regulator of telomere elongation helicase 1 (Rtel1)	Thera6—Ovaries	−0.341154
			Thera6—Testes	0.403238
			Thera2005—Ovaries	−0.0300314
			Thera2005—Testes	−1.69884
			B13005—Ovaries	−0.312243
			OBAT‐12003—Ovaries	−0.439944
			OBAT‐12003—Testes	−1.3714
GE21949	*Syx13*	Syntaxin 13 (Syx13)	Thera6—Ovaries	−0.700132
			Thera6—Testes	−0.793291
			Island Cascade‐1916—Ovaries	−0.105177
			Mainland Tai6—Ovaries	−0.57351
			Mainland Tai6—Female soma	−2.29854
			B13005—Ovaries	−0.391115
GE25331	*ctrip*	circadian trip (ctrip)	Thera6—Ovaries	0.0313342
			Thera6—Testes	0.0978823
			Thera2005—Ovaries	−0.823872
			Thera2005—Testes	−1.44162
			OBAT‐12003—Ovaries	−0.609531
			OBAT‐12003—Testes	−1.19085
GE16862	*mei‐9*	meiotic 9 (mei‐9)	Thera2005—Ovaries	−0.774595
			Thera2005—Testes	−1.60825
GE19943	*mus304*	mutagen‐sensitive 304 (mus304)	Mainland Tai6—Ovaries	−0.830693
			Mainland Tai6—Female soma	−4.03084
GE19033	*spel1*	spellchecker1 (spel1)	Thera6—Ovaries	0.0556472
			Thera6—Testes	−1.55863
			Thera2005—Ovaries	0.308494
			Thera2005—Testes	−0.0460433
			Island Cascade‐1916—Testes	−1.29778
			Mainland Tai6—Ovaries	−0.20007
			Mainland Tai6—Testes	−1.0472
			Mainland Tai6—Male soma	−1.45087
			Mainland Tai6—Female soma	−3.15695
			OBAT‐12003—Ovaries	0.352557
			OBAT‐12003—Testes	−1.2141
tacc	*tacc*	transforming acidic coiled‐coil protein (tacc)	Thera2005—Ovaries	0.217986
			Thera2005—Testes	−0.846894
			Thera2005—Male soma	1.01845
GE18360	*CG5181*	uncharacterized protein (CG5181)	Thera6—Ovaries	1.79065
			Thera6—Testes	1.5854

Island populations of both *D. santomea* and *D. yakuba* exhibited greater UV tolerance, with notable differences between sexes (Figures 3 and 4). To explore whether parallel changes in gene regulation contribute to the genetic response to shifting selective pressures, we compared the sets of genes showing significant expression differences between the two island populations and mainland Africa. We primarily observed independent changes across the two island populations, rather than shared solutions for UV tolerance in comparisons to mainland flies to both *D. yakuba* and *D. santomea*. However, each population contains multiple UV‐associated genes that have significantly different expression compared with mainland. *Pp2B‐14D* was upregulated in *D. santomea* males, while being down regulated in females [♂ 1.30916 and ♀ −0.296757]. We found *CycG*, a meiotic recombination DNA repair gene (Nagel et al. 2012), was downregulated in island *D. yakuba*. The gene *Rtel1* (annotated as CG4078) was implicated in DNA repair, as well as the maintenance of genomic and male germ line stability (Yang et al. 2021). This gene was found to be significantly differentially expressed in *D. santomea*. In all lines, among sexes, it was downregulated, except in the male line Thera6, where it was upregulated. *Syx13* was downregulated in all populations. The DNA damage response gene *ctrip* (Gaudet et al. 2011) was downregulated in all *D. santomea* except Thera6. *Mei‐9* is a known DNA nucleotide excision repair gene that was downregulated in only *D. santomea* Thera2005 [♀ −0.774595 and ♂ −1.60825]. *Mus304*, which interacts with *mei‐9*, was differentially expressed only in mainland *D. yakuba* populations, where it was downregulated [♀ −0.830693 and ♂ −4.03084], especially in males. The *spellchecker1* (*spel1*) gene, involved in post‐replication mismatch repair, was expressed across all populations but *D. santomea* line B1300.5. It was downregulated in all *D. yakuba* populations, except for female Cascade‐1916, where *spel1* was not significantly differentially expressed. Here, we observed that potential pathway‐level convergence in UV tolerance has arisen, rather than gene‐level convergence in gene expression changes. Such results suggest that independent genetic solutions arise even on short timescales in these *Drosophila* species, rather than common shared genetic solutions that might be facilitated by standing genetic variation.

However, one gene known to influence dimorphism shows a key example of genetic convergence for these two locally adapted populations. Sister‐of‐sex lethal (*ssx*) is an inhibitor of sex lethal (*sxl*) auto‐regulatory splicing (Moschall et al. 2019). *Ssx* was found in expression data from both island constituents, *D. santomea* and *D. yakuba*, but not in the mainland *D. yakuba* (see DAVID analysis Zenodo). Given these two species have a hybrid zone and are asymmetric in hybrid‐cross viability, we recommend further investigation in the future to determine whether this locus is affected by shared genetic solutions acquired via introgression.

Beyond indications of independent evolution across populations, notably, several genes are upregulated in female fly lines of *D. santomea* but are downregulated in males, consistent with sex‐specific effects that might influence sexual constraint during evolutionary change. We found that in *D. santomea* line Thera6, there was upregulation of transforming acidic coiled‐coil protein (*tacc*) for female ovaries and male soma but downregulated in the testes. Of our candidate DNA repair genes, *Pp2B‐14D*, *CG4078*, and *mei‐9* all reside on the X chromosome and were only found in *D. santomea*. Both *Pp2B‐14D* and *CG4078* were down regulated in the ovaries while also being upregulated in the testes. Within female *D. santomea*, *spel1* was upregulated, but was downregulated within males and all *D. yakuba*, both island and mainland populations. Finally, we found an uncategorized gene, *CG5181*, that is predicted to be involved in double‐stranded break repair (Barclay et al. 2014). This gene is predicted to be activated in response to ionizing radiation. It was exclusively found at significant upregulation in *D. santomea* line Thera6 [♀ 1.79065 and ♂ 1.5854]. In total, we identify 10 loci with gene expression changes across groups which are already known to influence UV tolerance. Here, these results recapitulate the observed sex‐specific differences observed in UV phenotypes. Variation in gene expression changes across strains confirms population genetic results that most mutations contributing to differentiation is segregating in populations, rather than fixed variation (Turner et al. 2021).

## 4 Discussion

### 4.1 Island Adaptation—Standing Variation and New Mutations

Evolutionary innovation can come from standing variation or from new mutation (Hermisson and Pennings 2005). Adaptation through standing variation is thought to occur quickly as phenotypic changes may be immediately beneficial under environmental changes (Goldschmidt 1982). New mutation, on the other hand, requires longer time periods to facilitate evolutionary change (Smith 1971; Hermisson and Pennings 2005). In theory, such pre‐existing variation could facilitate the immediate colonization of new niches exposed to ultraviolet radiation, even while new mutations may have accumulated over time to improve resistance further. Such a scenario aligns with Fisher's geometric model, where key innovations with a large initial effect enable the development of new phenotypes, followed by selection for multiple mutations that further refine these phenotypes (Orr 2005).

In our empirical testing, we find there is a strong signal that UV tolerance is greater on the island of São Tomé compared to the broadly distributed mainland Africa *D. yakuba*. In this study, we identify standing phenotypic variation for ultraviolet tolerance at low frequency in mainland populations that would be immediately available to facilitate shifts to higher altitude. The distribution of UV‐tolerant individuals in the mainland *D. yakuba* (ancestral population) is low; however, a subset of this population shows UV tolerance comparable to that of the island populations (Figure 3). These results suggest that standing genetic variation plays a role in local adaptation. While both island populations show enhanced UV tolerance compared to the mainland population, there remain key differences between the two species in that male and female *D. yakuba* differ in UV tolerance. Recoverability is much higher in *D. santomea* than in *D. yakuba*. *D. santomea* is better adapted given its lower morbidity in response to UV radiation (Figure 5). These differences in the phenotypic changes for these two species suggest that at least some portion of phenotypic divergence from the mainland is driven by variation that has evolved independently.

Colonization of *D. yakuba* on the island is approximately 400,000 years later than *D. santomea*, potentially offering time for new mutations to proliferate, with new genetic evidence of novel rearrangement‐induced changes that alter gene expression in three key UV tolerance genes (Turner et al. 2021). Whole genome gene expression data presented here suggest that novel expression patterns have emerged for multiple genes in both island populations, again with independent genetic responses via new mutation (Table 4). Together, these genetic and phenotypic results lead us to suspect that while standing variation may have been present to allow niche colonization, new mutations have also contributed to the refinement of phenotypes during local adaptation at higher UV exposure.

### 4.2 Genetic Parallels Across Species

Ultraviolet tolerance has evolved in parallel in multiple populations and species of *Drosophila* (Pool and Aquadro 2007; Zhao et al. 2015; Svetec et al. 2016). If few pathways exist to facilitate phenotypic change, we may expect to observe higher rates of convergence at the genetic level when we observe convergent changes across phenotypes (Stern 2013). However, if there exist multiple independent genetic solutions, genetic convergence may be less common. As an initial step toward connecting genotype to phenotype, we identify multiple UV tolerance genes that show gene expression changes in *D. santomea*. Prior experiments have found many of the same genes or similar pathways identified in our own genetic analysis of UV resistance. The genes *phr*, *Dmp8/TTDA*, and *mei‐9* are potential mutants involved in ultraviolet resistance, demonstrating strong photo‐repair activity in the *Drosophila* Trichothiodystrophy model (Boyd and Harris 1987; Yildiz et al. 2004; Aguilar‐Fuentes et al. 2008). Neither *phr* nor *Dmp8*/*TTDA* are found to have any significant up/down regulation in *D. santomea* or *D. yakuba* on the island. The lack of concordance suggests that *Drosophila* from São Tomé may utilize a different genomic mechanism for UV resistance, rather than relying on gene‐level convergent evolution as seen in *D. melanogaster* .

At certain loci, however, there is potential for parallel changes in gene expression when compared to other systems. Our functional analysis has overlapping genes with the UV gene list from Svetec et al. (2016) at *CycG*, *mei‐9*, *mus304*, and *spel1*. In this study, Syntaxin 13 (*Syx13*) is significantly downregulated across all populations. Turner et al. (2021) found three more candidate genes that have significant rearrangements that are annotated as UV resistance genes, which include *Parp*, *Victoria*, and *spel1*. Both *Parp* and *spel1* have significant expression changes in *D. santomea* (Turner et al. 2021). Further evidence of a genetic factor that may mitigate damage from ultraviolet radiation is the gene *Grapes* (*grp*), another DNA repair gene, which is found only in the *D. santomea* group (see DAVID analysis gene list on Zenodo). These findings provide insights into how *D. santomea* may cope with ultraviolet radiation beyond protective phenotypes like increased melanin production, contrasting with other high‐altitude organisms (Goldman 1947, Reguera et al. 2014, Harris et al. 2013; de Souza et al. 2020; Ulbing et al. 2019).

### 4.3 Connection to Environmental Stressors

During this experiment, we ran distribution models with MaxEnt 3.4.1 (Phillips et al. 2017) from locality data taken from Comeault and Matute (2021). Though, besides *D. santomea* being more UV tolerant than *D. yakuba*, annual geographic UV radiation data from WorldClim 2.1 potentially show extrinsic sources for the lack of melanistic patterning at higher altitude. Lachaise et al. 2000 described the habitat of *D. santomea* residing in mist forests. These mist forests may have less UV exposure than disturbed areas or at least less solar radiation. Current evidence from this study illustrates that *D. santomea* are in more UV exposed areas. Pairing this detailed geographic variation for UV stress allows us greater precision as we evaluate and interpret phenotypic screens in controlled lab settings.

The newly developed apparatus for UV stress assays contributes further to our understanding of this known phenotypic stressor in nature. With these high‐throughput phenotyping scans that include recorded data for post‐experiment validation, we improve the reproducibility and rigor for UV studies. We can control confounding variables such as temperature during longer acute UV exposure. This innovation allows us to survey the response to acute UV stress, with both morbidity and mortality for multiple strains from different populations. While morbidity may allow for short‐term survival in controlled environments, limited activity could become lethal in the wild due to factors such as predation, secondary desiccation, or invasion by pathogens and parasites. Other stressors may also lead to death during periods of inactivity in natural environments. Our surveys of this key phenotype pave the way for new studies on the potentially complex interactions between UV stress and survivorship in natural systems.

### 4.4 Sexual Dimorphism for UV Tolerance

Sex‐specific phenotypes can free populations from evolutionary tradeoffs in reproductive strategies and behavioral risks between males and females (Darwin 1871; Fisher 1931; Parker 1979; Lande 1980). Decoupling phenotypic effects across sexes frees evolution from pleiotropic constraints to achieve sex‐specific optima (Trivers 1972; Lande 1980; Albert and Otto 2005). Our results are consistent with these models for the evolution of dimorphic traits, as we observe that sex has a large role in UV tolerance. Even our control population of mainland Africa, *D. yakuba*, the females are more UV tolerant than to males; however, this result is not significant from statistical tests when conditioning on population ancestry, only significant when analyzing sex. Given that females do better as a whole and do better than males within the island *D. yakuba* population, females could have a sex‐linked factor that leaves them more well adapted to UV exposure. The presence of expression changes for X‐linked loci with connections to UV repair strongly suggests a genetic mechanism that evolves differently for the two sexes, contributing to dimorphic traits. When observing recovery rates across population and sex, overall *D. santomea* demonstrates a higher recovery rate and greater activity over time compared to other populations. Female island *D. yakuba* consistently show the highest levels of activity across all tested groups (Figure 5). Females on the island are more UV tolerant and recover better than males in *D. yakuba*. The genetic basis for these changes across groups aligns with these sex‐specific effects, as several loci are found on sex chromosomes or exhibit sex‐specific differences in gene expression changes for multiple genes. Many phenotypes show sexual dimorphism in this species complex (Llopart et al. 2002, 2005a, 2005b; Matute et al. 2009). *D. santomea* follow Haldane's rule where the heterogametic sex is inviable (Coyne 1985.) Our assays on UV tolerance further confirm sex‐specific evolutionary changes in this key species complex and point to even more widespread dimorphism than was previously reported. Future work on the genomic underpinnings of UV tolerance within the system studied here may reveal even more complete explanations for how these key phenotypes have evolved during habitat shifts. Here, the use of sex as a biological variable reveals differing modes of adaptation in the face of environmental change, necessary to understand the full complexity of evolutionary trajectories in nature. As new studies aim to uncover the phenotypic and genetic basis of adaptive changes, sex‐specific assays are likely to hold the key to understanding how evolutionary constraint shapes variation in nature.

### 4.5 Conclusion

In this study, we have clarified that, of the populations surveyed, *D. santomea* residing at higher elevations, exposed to more ultraviolet radiation, do indeed tolerate UV stress better than *D. yakuba*. Island *D. yakuba* populations tolerate ultraviolet radiation better than those of their mainland constituents. Sex‐specific effects play a strong role in UV tolerance, where females have a higher tolerance to UV radiation than males in all populations and X‐linked factors are associated with changes in gene expression. Female island *D. yakuba* are even more UV tolerant than *D. santomea*, both in male and female sexes of the species, curiously. For our study, we also constructed an easily reproducible UV exposure apparatus with commonly found lab equipment and provide enclosure schematics for other researchers to use for future experiments (Supporting Information). Future research on the genetic basis of UV tolerance in this system could provide valuable insights into the molecular mechanisms underlying responses to environmental stressors. This system, which does not follow current observations of altitude‐induced melanism offers new avenues to study alternative paths to adaptation in nature. Further research should be conducted on the genes potentially allowing for less pigmented *D. santomea* to occur at higher altitudes than other *Drosophila*, including their more melanistic congenere.

## Author Contributions

**James E. Titus‐McQuillan:** conceptualization (lead), data curation (lead), formal analysis (lead), investigation (equal), methodology (lead), validation (lead), visualization (lead), writing – original draft (lead), writing – review and editing (equal). **Brandon A. Turner:** formal analysis (supporting), investigation (supporting), methodology (supporting), validation (supporting), writing – review and editing (supporting). **Rebekah L. Rogers:** formal analysis (supporting), funding acquisition (lead), investigation (equal), methodology (equal), resources (equal), supervision (equal), writing – original draft (supporting), writing – review and editing (equal).

## Conflicts of Interest

The authors declare no conflicts of interest.

## Benefit Sharing

By recording our trials and releasing them for review, we allow for further investigations into questions not proposed or conceptualized by this team. There is potentially a wealth of knowledge to gain from these recorded trials that may expand into other studies.

## Supporting information


Data S1.

## Data Availability

All genetic data was procured from Turner et al. (2021). Sequence data is stored and available under SRA PRJNA764098, PRJNA764689, PRJNA764691, PRJNA764693, PRJNA764695, PRJNA764098, PRJNA269314. All trials have associated video recordings along with data and code stored on Zenodo DOI:10.5281/zenodo.13894098. Statistical code and data is also availble on GitHub‐ https://github.com/jemcquillan/UV_Assay_Sao_Tome.
