# Supplementary material for: Sex‐Specific Ultraviolet Radiation Tolerance Across Drosophila
Source: Ecol Evol. 2025 Feb 25;15(2):e70985. doi: 10.1002/ece3.70985 (PMC11855014; doi:10.1002/ece3.70985)
Supplement: Supplementary file 1 — Data S1. [file ECE3-15-e70985-s001.zip › Supp.mat.pdf]

Supplementary Materials:

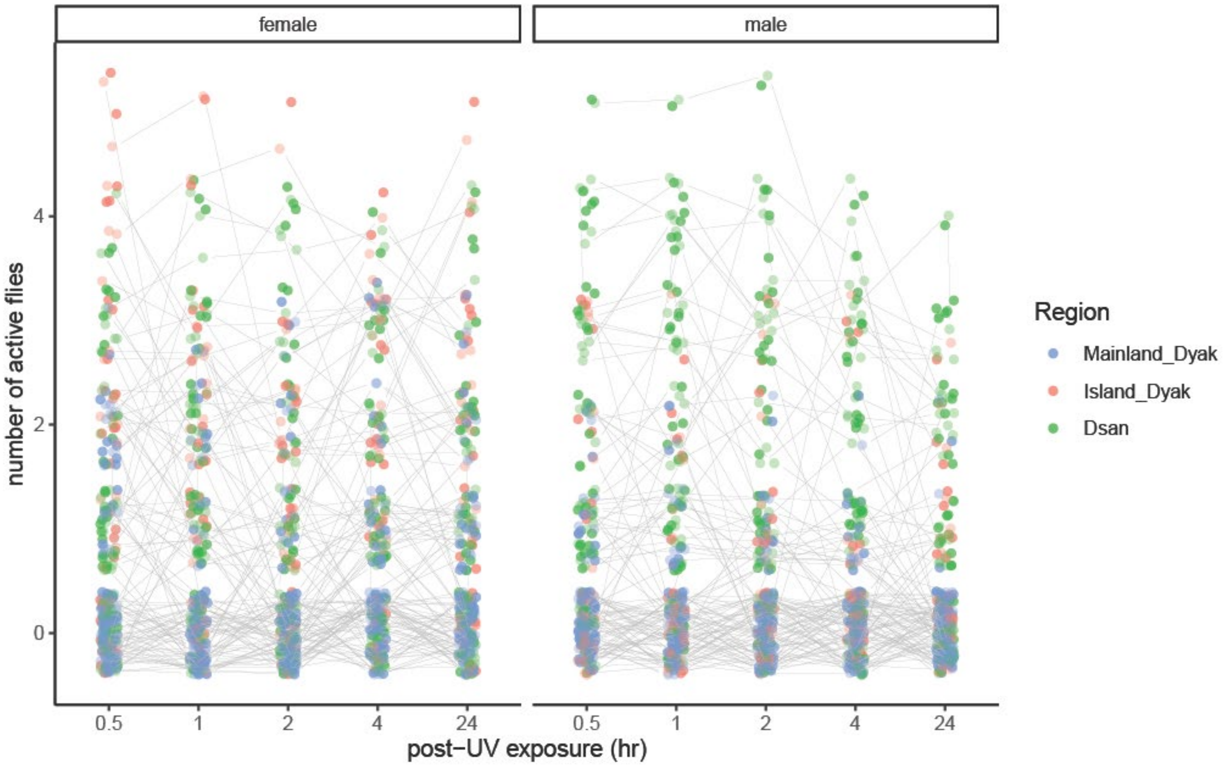

Supp. 1: Active fly counts post UV exposure split by sex.

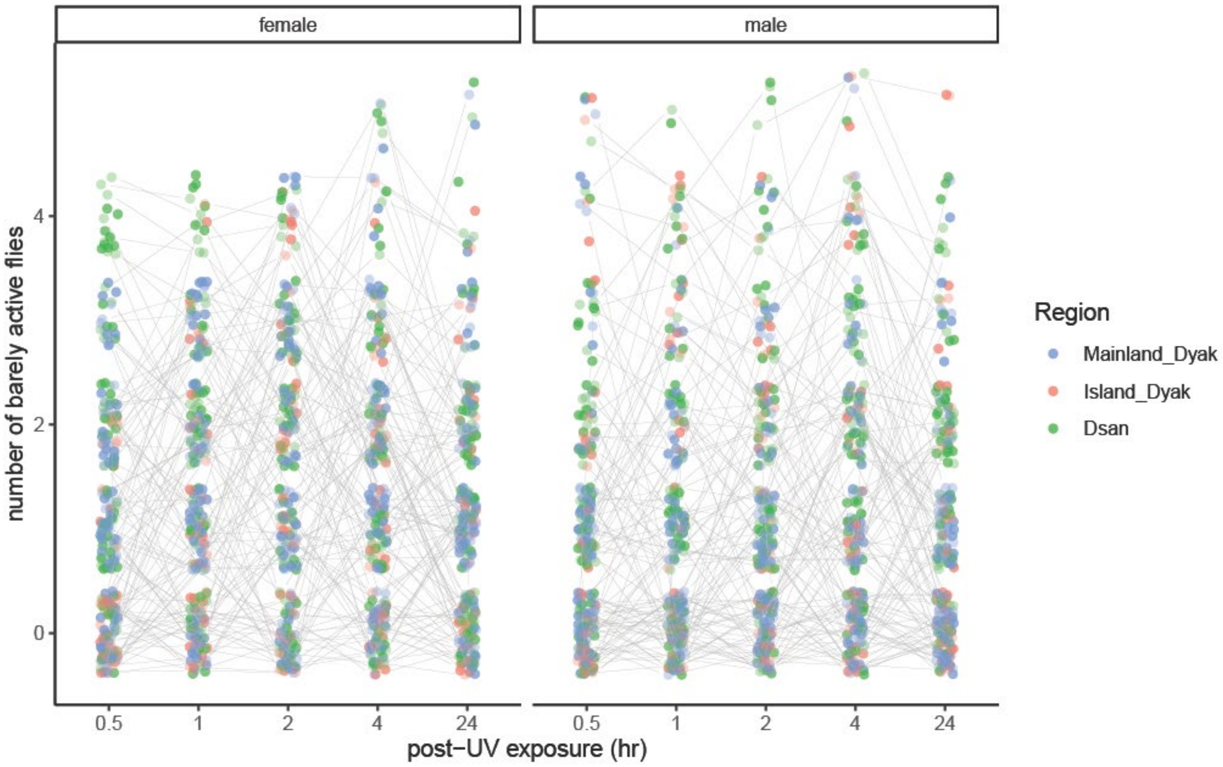

Supp. 2: Barely active counts post UV exposure split by sex.

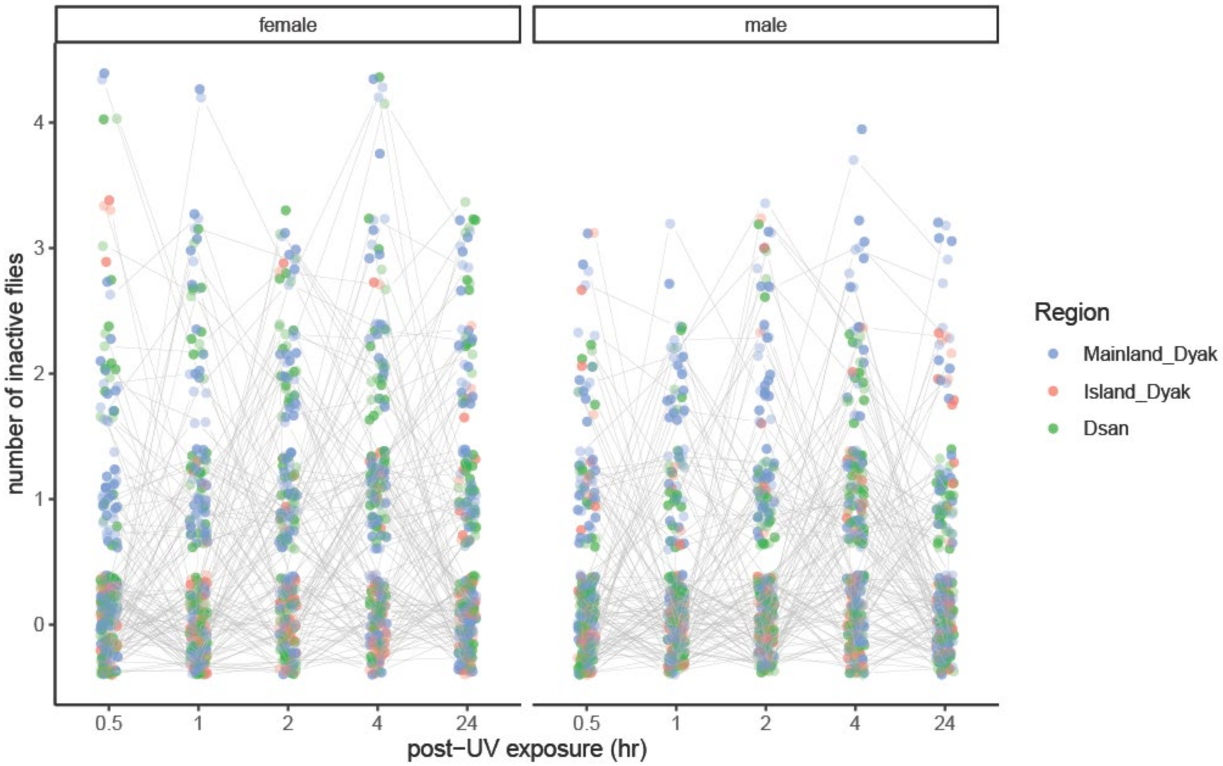

Supp. 3 Inactive counts post UV exposure split by sex.

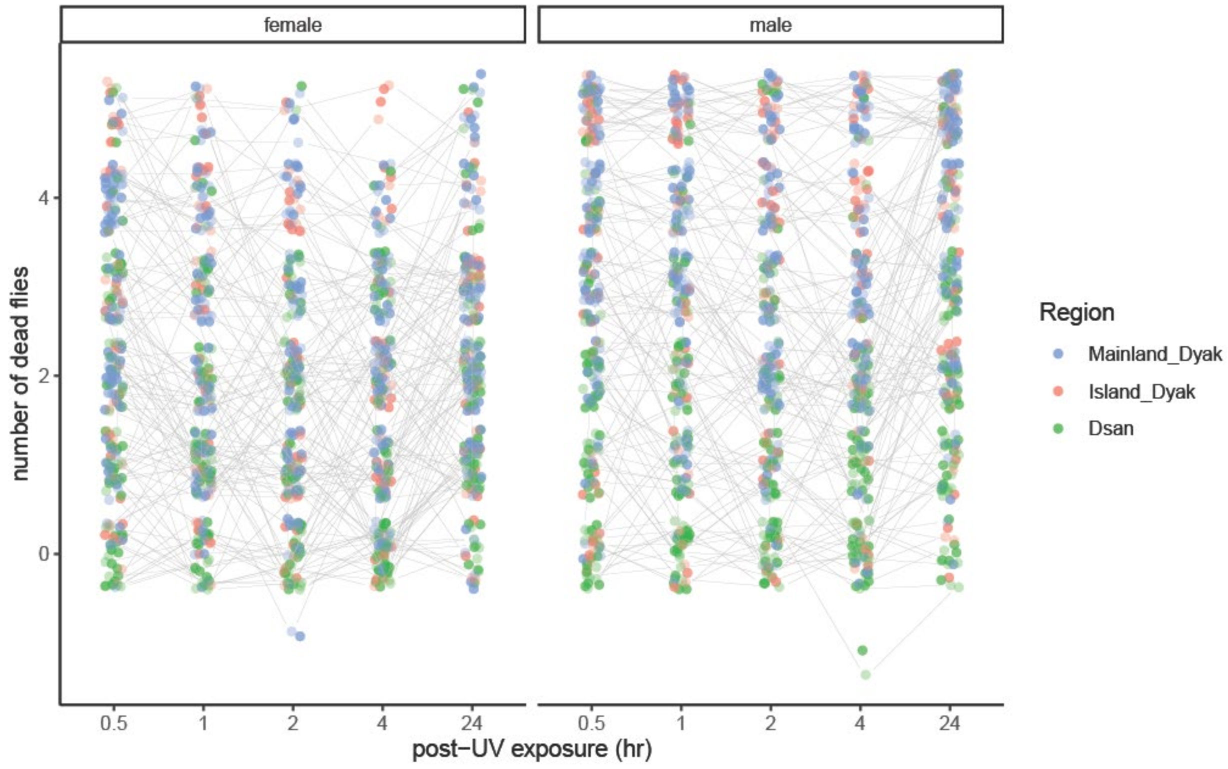

Supp. 4: Dead counts post UV exposure split by sex.

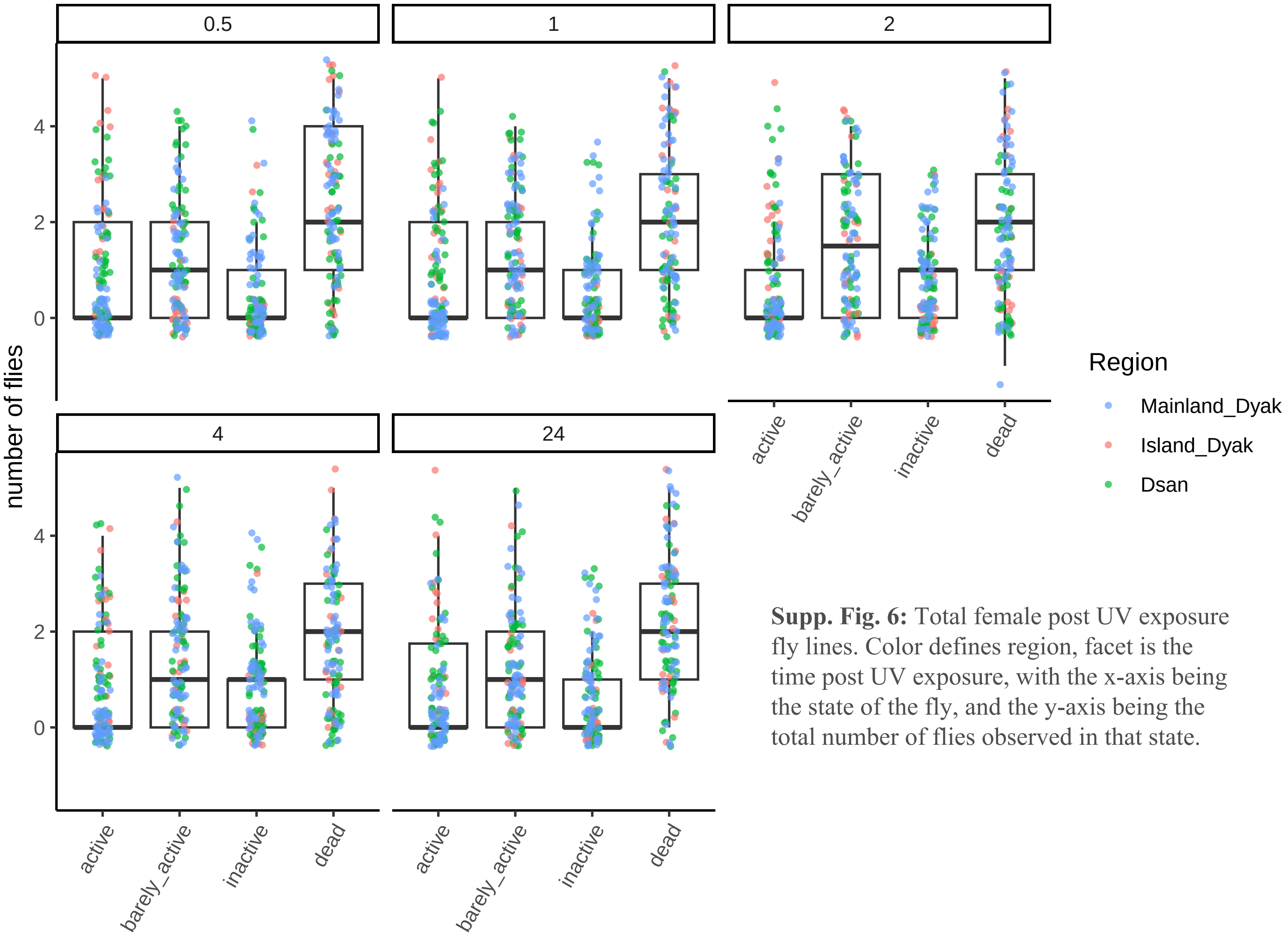

Supp. Fig. 6: Total female post UV exposure fly lines. Color defines region, facet is the time post UV exposure, with the x-axis being the state of the fly, and the y-axis being the total number of flies observed in that state.

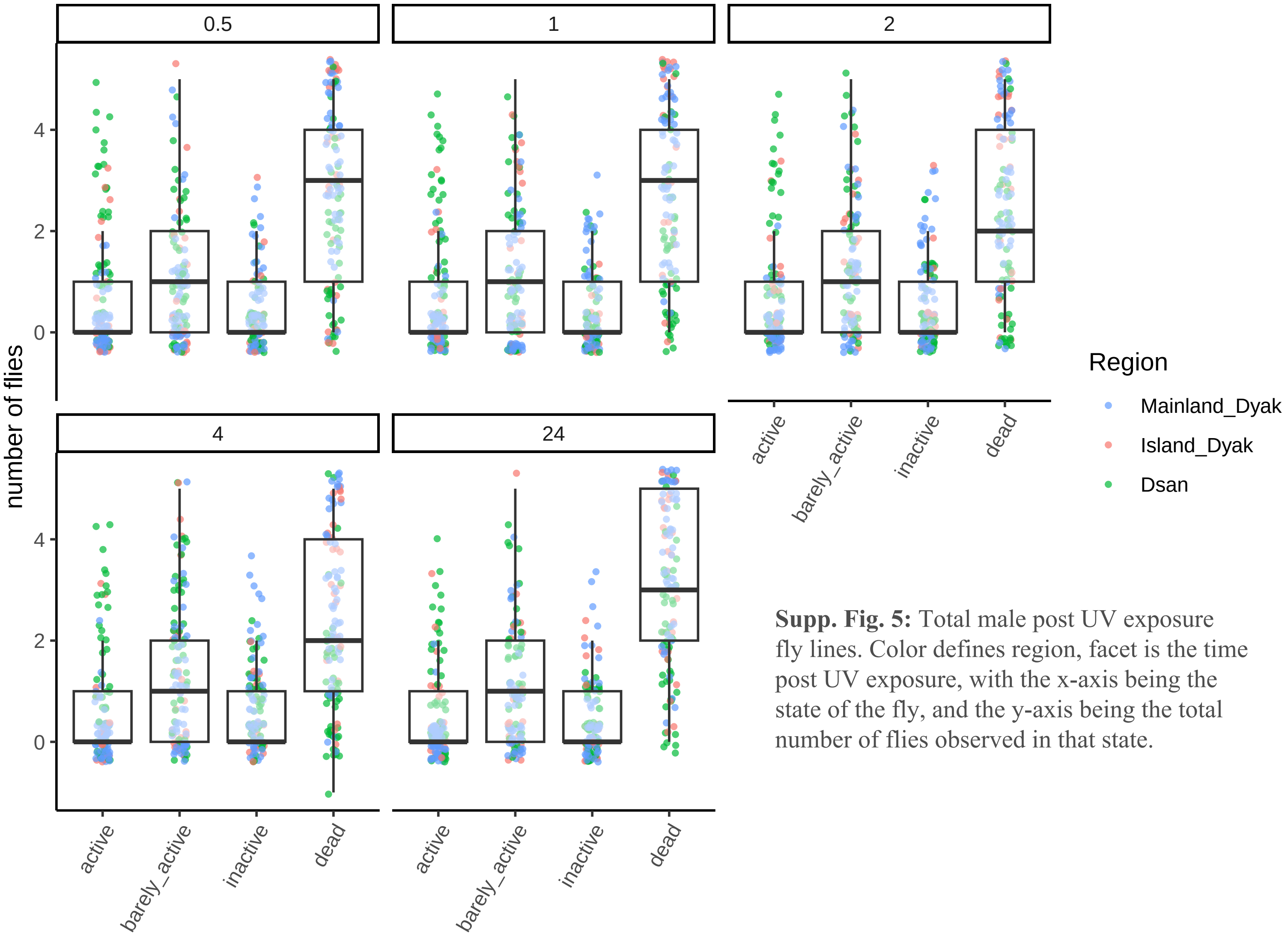

Supp. Fig. 5: Total male post UV exposure fly lines. Color defines region, facet is the time post UV exposure, with the x-axis being the state of the fly, and the y-axis being the total number of flies observed in that state.
