# Supplementary figures and images for: Sex‐Specific Ultraviolet Radiation Tolerance Across Drosophila
Source: Ecol Evol. 2025 Feb 25;15(2):e70985. doi: 10.1002/ece3.70985 (PMC11855014; doi:10.1002/ece3.70985)

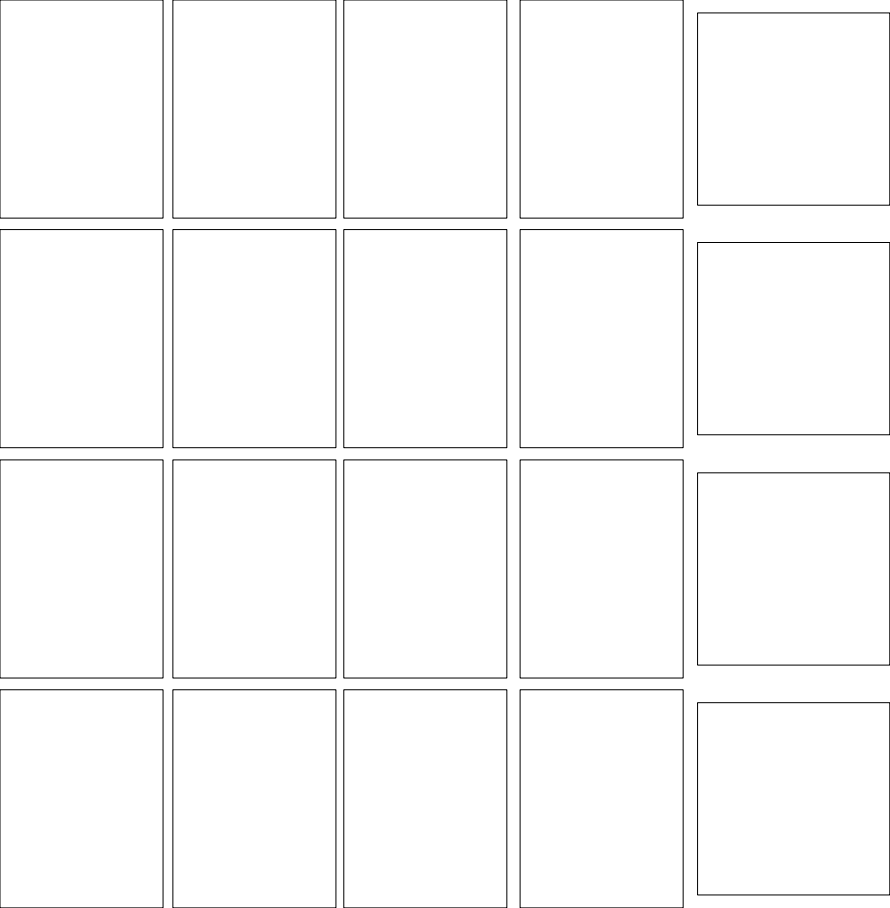

Supplement: Supplementary file 1 — Data S1. [file ECE3-15-e70985-s001.zip › Supp.mat.dros.acrylic_enclosure_schematic.pdf]
